# Effect of dietary additives on intestinal permeability in both *Drosophila* and a human cell co-culture

**DOI:** 10.1242/dmm.034520

**Published:** 2018-11-28

**Authors:** Matthew T. Pereira, Mridu Malik, Jillian A. Nostro, Gretchen J. Mahler, Laura Palanker Musselman

**Affiliations:** 1 Department of Biological Sciences, Binghamton University, Binghamton, New York 13902, USA; 2 Department of Biomedical Engineering, Binghamton University, Binghamton, New York 13902, USA

**Keywords:** Gut barrier function, Intestinal permeability, Alkaline phosphatase, *Drosophila*

## Abstract

Increased intestinal barrier permeability has been correlated with aging and disease, including type 2 diabetes, Crohn's disease, celiac disease, multiple sclerosis and irritable bowel syndrome. The prevalence of these ailments has risen together with an increase in industrial food processing and food additive consumption. Additives, including sugar, metal oxide nanoparticles, surfactants and sodium chloride, have all been suggested to increase intestinal permeability. We used two complementary model systems to examine the effects of food additives on gut barrier function: a *Drosophila in vivo* model and an *in vitro* human cell co-culture model. Of the additives tested, intestinal permeability was increased most dramatically by high sugar. High sugar also increased feeding but reduced gut and overall animal size. We also examined how food additives affected the activity of a gut mucosal defense factor, intestinal alkaline phosphatase (IAP), which fluctuates with bacterial load and affects intestinal permeability. We found that high sugar reduced IAP activity in both models. Artificial manipulation of the microbiome influenced gut permeability in both models, revealing a complex relationship between the two. This study extends previous work in flies and humans showing that diet can play a role in the health of the gut barrier. Moreover, simple models can be used to study mechanisms underlying the effects of diet on gut permeability and function.

This article has an associated First Person interview with the first author of the paper.

## Abstract (precis)

**Summary:** Sugar and other food additives can inhibit gut barrier function in both human cell co-cultures and *Drosophila melanogaster.*

## INTRODUCTION

The gastrointestinal (GI) tract is the largest surface within the human body exposed to the external environment, and the GI epithelium is composed of a single continuous layer of cells. Consisting of all structures between the mouth and the anus, the GI tract is made up of the esophagus, stomach and intestines. The main function of the GI tract is to act as a selective barrier that protects against potentially harmful luminal content such as foreign antigens and toxins, while simultaneously allowing nutrients and water to pass through the epithelium and enter the circulation. The small intestine is the primary site of nutrient absorption (DeSesso and Jacobson, 2001), which occurs in one of two manners. Materials can pass transcellularly, across the membranes of cells of the epithelium, or paracellularly, in between these cells. Transcellular transport is selective and is mediated by passive or active transporters found on the luminal and/or basal surfaces of the epithelium. In vertebrates, paracellular transport is mediated by structures connecting epithelial cells together, called tight junctions. Tight junctions form the foundation of the paracellular barrier (Ma and Anderson, 2006) and vary in composition depending on their position within the epithelium (Chiba et al., 2007). The paracellular barrier in *Drosophila* and other invertebrates is made up of septate junctions, which are functionally analogous to tight junctions (Izumi and Furuse, 2014; Jonusaite et al., 2016). Disruption of intestinal barrier function by loss of integrity of the paracellular barrier leads to luminal contents spilling into the surrounding tissues, inflammation and potentially tissue damage (Ma and Anderson, 2006). Abnormal intestinal permeability has been observed in various diseases, including type 2 diabetes, Crohn's disease, celiac disease and irritable bowel syndrome (Arrieta et al., 2006; Farhadi et al., 2003; Fasano and Shea-Donohue, 2005; Harris et al., 1992; Sandek et al., 2008). The prevalence of these diseases has been rising (Selmi, 2010) along with increasing industrial food processing and consumption of food additives (Manzel et al., 2014) such as glucose (Fihn et al., 2000; Nusrat et al., 2000; Turner et al., 2000; Yu et al., 2013), salt (Madara and Carlson, 1991; Madara et al., 1987, 1993; Pappenheimer and Volpp, 1992), surfactants (also called emulsifiers) (Hamid et al., 2009; Mine and Zhang, 2003; Sha et al., 2005) and nanoparticles (Das and Chaudhury, 2011; Loo et al., 2012; Lv et al., 2013; Saremi et al., 2013; Zhang et al., 2014) such as titanium dioxide (TiO_2_). Therefore, we were interested in testing whether these additives could affect intestinal permeability using model systems.

Intestinal barrier dysfunction has been described in mammalian (Katz et al., 1987; Kirkwood, 2004) and *Drosophila melanogaster* (called *Drosophila* hereafter for simplicity) models (Biteau et al., 2008; Choi et al., 2008; Park et al., 2009; Rera et al., 2011). The *Drosophila* gut is regionalized and expresses many of the same proteins as the human gut (Buchon and Osman, 2015). Under some conditions, adult fly guts carry out both transcellular and paracellular transport of macromolecules (MacMillan et al., 2017; Shanbhag and Tripathi, 2005). The fly gut, however, has a chitinous peritrophic matrix that regulates interactions between the gut epithelium and its contents (Kuraishi et al., 2011), and the fly seems to have fewer species in its microbiome when compared with humans (Han et al., 2017). In both flies and humans, increased permeability across the intestinal epithelium is tightly correlated with aging. Previous studies have also shown that chronic inflammation, type 2 diabetes, reduced metabolic stores and increased bacterial load are also associated with aging in both humans (Cheng et al., 2013; Chung et al., 2009; López-Otín et al., 2013; Lusis et al., 2008) and *Drosophila* (Landis et al., 2004; Morris et al., 2012; Pletcher et al., 2002; Ren et al., 2007; Rera et al., 2012). Diet also affects intestinal barrier permeability (Rapin and Wiernsperger, 2010; Rera et al., 2012) and may play a direct or indirect role by changing the microbiome and/or the rate of aging.

The GI tract harbors commensal, opportunistic and sometimes pathogenic bacteria, which interact directly and indirectly with GI cells. When the intestinal barrier is compromised in patients suffering from inflammatory bowel disease (IBD), the microbiota is altered compared to healthy individuals (Lane et al., 2017; Round and Mazmanian, 2009). Flies with gut barrier dysfunction also have shifts in the microbiome (Clark et al., 2015), and there is evidence that the microbiome can influence lifespan (Brummel et al., 2004; Clark et al., 2015). Probiotic bacteria can also play a protective role in IBD patients (O'Flaherty and Klaenhammer, 2012). *Lactobacillus rhamnosus*, a gram-positive bacterium, has been shown to reduce intestinal permeability in IBD patients (Francavilla et al., 2010) and may reduce bacterial lipopolysaccharide (LPS)-associated gut inflammation (Zhang et al., 2006).

Intestinal alkaline phosphatase (IAP) is a gut mucosal defense factor exclusively produced in the small intestine that detoxifies LPS by dephosphorylation of the lipid A moiety (Beumer et al., 2003; Poelstra et al., 1997), thereby protecting against LPS-induced inflammation (Goldberg et al., 2008). IAP also regulates bicarbonate secretion and duodenal surface pH (Akiba et al., 2007; Mizumori et al., 2009), and regulates intestinal lipid absorption across the enterocyte apical membrane (Narisawa et al., 2007, 2003). Studies conducted in Caco-2 cells and C57BL/6N mice show that IAP expression reduces inflammation and enhances tight junctions by decreasing the expression of vascular endothelial growth factor, which is a cytokine that may increase intestinal permeability by reducing the levels of dephosphorylated ERK, and downregulating claudin-2, which is a cation-channel-forming protein (Liu et al., 2016; Wang et al., 2015). These regulatory and anti-inflammatory effects aid in maintaining intestinal barrier function and preventing bacterial invasion across the gut mucosal barrier, leading several investigators to propose that IAP contributes to intestinal barrier integrity (De Lisle et al., 2011; Economopoulos et al., 2016; Kaliannan et al., 2013; Liu et al., 2016; Molnár et al., 2012). Caco-2 barrier function was enhanced with IAP supplementation, which resulted in upregulated zonula occludens (ZO)-1 and ZO-2 expression, and ameliorated LPS-induced increases in permeability (Liu et al., 2016). This suggests a positive correlation between IAP expression, tight-junction functionality and intestinal permeability.

We used two complementary models to analyze how dietary additives control gut permeability. In the *Drosophila in vivo* model, we analyzed the effects of feeding high sucrose, TiO_2_ nanoparticles (NP), the emulsifier TWEEN 20 and sodium chloride (NaCl or table salt) on intestinal integrity. A second Caco-2 and HT29-MTX cell-based *in vitro* intestinal epithelium model was used to study the effects of high glucose and TiO_2_ NP within a food matrix on intestinal membrane integrity. Caco-2 cells, derived from colonic epithelial adenocarcinoma cells, have the ability to polarize and differentiate into an enterocyte-like epithelial barrier, including the expression of microvilli and tight junctions. Caco-2 cells also secrete surfactant-like particles (Engle et al., 2001) that are rich in IAP (Eliakim et al., 1989). The HT29-MTX cell line is derived from human colonic adenocarcinoma cells that are adapted to methotrexate (MTX). This transforms the cells into differentiated, goblet-like, mucus-secreting cells (Lesuffleur et al., 1990). HT29-MTX cells represent goblet cells and form a protective mucus layer on top of the Caco-2 cell layer (Lesuffleur et al., 1995). The Caco-2/HT29-MTX co-culture system has been used previously to model the small intestine and to study the role of NP ingestion on intestinal function (Guo et al., 2017, 2018; Mahler et al., 2012; Richter et al., 2018).

Using both models, we observed significant effects of food additives on intestinal barrier function, with high sugar producing the largest differences. To explore the mechanisms underlying the observed changes in intestinal physiology, we used physiological, biochemical and immunohistochemical analyses. Sugar had the most dramatic effects, and sugar-dependent microbial influences were also observed in each model. Together, these *in vivo* and *in vitro* models serve as sensitive platforms for testing and understanding the complex effects of the microbiome and dietary additives on intestinal permeability.

## RESULTS

### Dietary additives affect intestinal integrity *in vivo*

Rera et al. developed a non-invasive *in vivo* assay to test intestinal integrity in *Drosophila* using a nonabsorbable blue food dye [The United States Federal Food, Drug, and Cosmetic Act (FD&C) blue dye #1] (Rera et al., 2011, 2012). The assay has been dubbed the ‘Smurf assay’ after the characteristic blue look of flies exhibiting intestinal integrity loss (Rera et al., 2011, 2012), hereinafter referred to as ‘Smurfs’. Using this assay, it has been shown that intestinal barrier dysfunction increases with age and also depends upon the diet under some conditions (Clark et al., 2015; Rera et al., 2012). We used the Smurf assay to test the effects of different diets on intestinal barrier dysfunction. Young adult flies of the common laboratory strain *w^1118^* were transferred to diets containing FD&C blue dye #1, which can be observed in the gut of a live fly shortly after ingestion (Rera et al., 2011, 2012). In healthy flies, this blue dye is restricted to the GI tract and can be observed in the gut and on the proboscis (Fig. S1A). With an increase in intestinal permeability, blue dye can be seen outside of the gut, spilling into the abdomen, thorax, head and legs of the fly (Fig. S1B). Our experimental diet is more nutrient-dense than cornmeal-based fly diets (Musselman et al., 2011), and females typically eat more than males (Wong et al., 2009). We found that many males do not eat enough to observe dark blue guts, especially in aged flies, making it difficult to detect Smurfing. Therefore, males had limited utility and so we eliminated them from our study paradigm early in this project. Only female flies were used to quantify gut permeability and females were assumed to have mated after housing with males for 3 days. Quantification was done for the entire adult lifespan.

Controls were fed a control diet (0.15 M or 5% sucrose) and experimental groups consumed either a: high-sugar (HS; 1.0 M or 34% sucrose), 50 ppm TiO_2_, 500 ppm TiO_2_, 1% NaCl or 1% TWEEN 20 diet. The concentrations of additives were chosen to correspond to a physiologically realistic human diet. Sugar-sweetened beverages contain up to 11% sugar (Ventura et al., 2011), with candy often containing more than 50% sugar (Welsh et al., 2011), so these concentrations are representative of what some individuals experience. Chronic HS feeding also produces insulin resistance in adult flies (Na et al., 2013), and flies with a compromised gut barrier exhibit impaired insulin signaling, with a reduced degree of Akt phosphorylation, despite increased insulin receptor expression (Rera et al., 2012). We also tested a metal oxide nanoparticle, the food additive TiO_2_, in both models. The United States Food and Drug Administration allows the use of up to 1% TiO_2_, although lower concentrations are typically used. One study measured the TiO_2_ content of over 100 different food items, which ranged from 7.7×10^−5^ to 0.359% with an average of 0.0579%, 36% of which is nano-sized (Weir et al., 2012). The physiological concentrations we used *in vivo* (50 ppm/0.005% and 500 ppm/0.05%) therefore fall within the range of TiO_2_ NP concentrations found in common orally administered products. In flies, we also used salt and an emulsifier. The 1% sodium chloride concentration was based on salt content up to 3.5% by weight found in processed foods (Webster et al., 2010). The FDA allows up to 4.5% of emulsifier by weight, making our concentration of 1% TWEEN 20 fall within the range of emulsifier concentrations experienced in human diets (U.S. Food and Drug Administration, 2017).

**Fig. 1.**
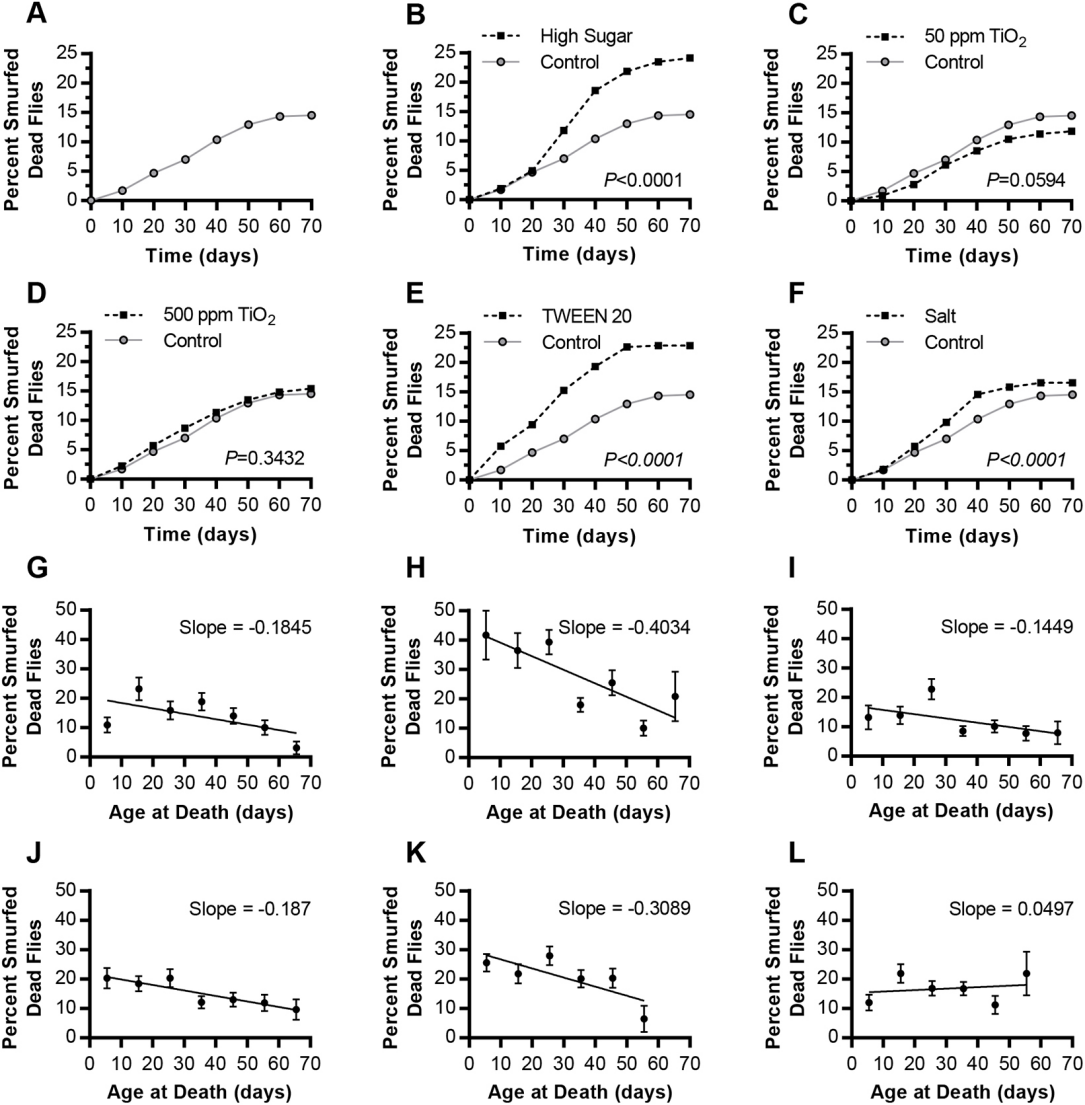
**The Smurf phenotype shows increased blue-dye leakage from the gut into the body cavity with dietary supplementation.** Dead flies were rinsed and visually scored for the Smurf phenotype. (A-F) The cumulative percentage of flies Smurfed at death. (A) Control diet (0.15 M sucrose; *n*=945). (B) High-sugar (HS) diet (1.0 M sucrose; *P*<0.0001, χ^2^ 17.27, d.f.=1; *n*=797). (C) 50 ppm TiO_2_ diet (*P*=0.0594, χ^2^ 3.554, d.f.=1; *n*=999). (D) 500 ppm TiO_2_ diet (*P*=0.3432, χ^2^ 0.8986, d.f.=1; *n*=1189). (E) 1% TWEEN 20 diet (*P*<0.0001, χ^2^ 55.55, d.f.=1; *n*=937). (F) 1% salt diet (*P*<0.0001, χ^2^ 16.59, d.f.=1; *n*=949). Each experimental diet was compared with the control diet. *P*-values calculated using the Mantel–Cox log-rank test in A-F. (G-L) The non-cumulative data representing the percentage of flies who were Smurfed upon death. (G) Control diet. (H) HS diet. (I) 50 ppm TiO_2_ diet. (J) 500 ppm TiO_2_ diet. (K) 1% TWEEN 20 diet. (L) 1% salt diet. Error bars represent s.e.m. and the best-fit slope was calculated using a linear regression.

Smurfing was quantified in live flies (Fig. S1) as they aged, as well as in each fly upon death (Fig. 1). This allowed us to quantify the abundance of Smurfs at different time intervals, counting each fly multiple times across its lifespan. We also quantified the overall Smurfing of a population by recording each fly's condition upon death, calculated by dividing the cumulative number of dead Smurfs by the total amount of dead flies on that diet. HS feeding increased gut permeability when compared to control diets, and did so to a higher degree than every other diet tested with a cumulative 24.09% of flies Smurfing before death (Fig. 1). Both TWEEN 20 and NaCl increased intestinal barrier permeability as measured by Smurfing, compared with control diets, whereas TiO_2_ feeding had relatively weak effects on intestinal barrier function (Fig. 1A,C-F). More than 40% of HS-fed flies who died young were Smurfed, whereas around 20% of old, dead HS flies were Smurfed, resulting in a decrease in Smurfing rate over time (Fig. 1G,H). Five of six diets resulted in a decrease in rate of Smurfing over time (Fig. 1G-L), which may result in part from reduced feeding in aged flies, leading to reduced assay sensitivity. HS, which had the highest overall Smurfing rates, also had the most negative slope, with the highest Smurfing rates in flies that died young. The differences between early- and late-compromised gut barrier integrity can also be seen by observing the live fly Smurfing over time (Fig. S1). A 1% TWEEN 20 diet resulted mostly in early-age Smurfing in live flies, while a 1% salt diet resulted in Smurfing predominantly after 3 weeks of age, and a HS diet resulted in Smurfing in both young and old live flies (Fig. S1C-K).

### Sugar content affects intestinal integrity *in vitro*

To model gut permeability *in vitro*, we used a co-culture model of Caco-2 cells and HT29-MTX cells seeded onto semi-permeable membranes (Mahler et al., 2009). In this model, we tested intestinal permeability using the dye Lucifer Yellow (LY) (Hidalgo et al., 1989). The cell monolayer was treated with LY upon exposure to food additives. LY passively or transcellularly passed through the cell monolayer and was collected from the basolateral chamber of the cell model for a fluorescence assay. The amount of LY collected in the bottom chamber increases as permeability across the cell monolayer increases.

**Fig. 2.**
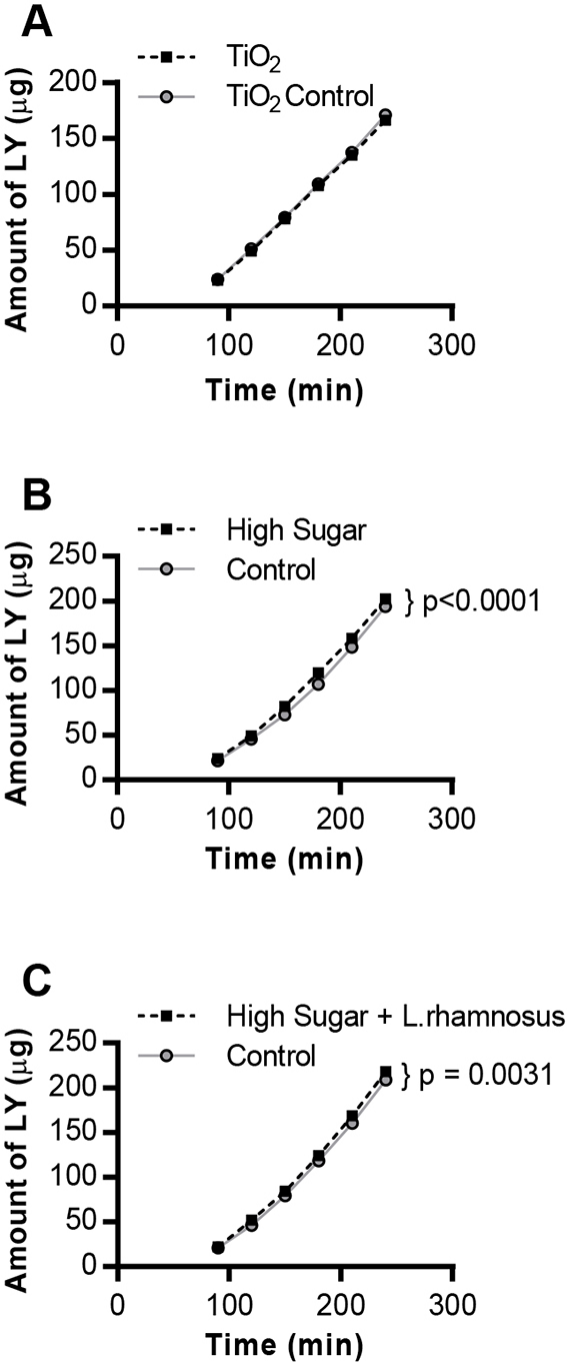
**Food-additive exposure increases permeability in a human gut cell co-culture model.** Lucifer Yellow (LY) permeability following exposure of the Caco-2/HT29-MTX cell monolayer to: (A) TiO_2_ or TiO_2_-free (control) food digest for 4 h (*n*=6), (B) control (5 mM glucose+20 mM mannitol) or high-glucose (25 mM; HS) culture medium for 2 h (*n*=12, *P*<0.0001), or (C) control or HS culture medium for 24 h (*n*=12, *P*=0.0031). *F*-test-generated *P*-values denote significant differences between treatments and controls according to a non-linear fit for a linear model for each sample set.

Concentrations of TiO_2_ for our *in vitro* model were chosen based on the fact that the average 70 kg adult consumes 70 mg of TiO_2_ (25 mg of which is nano-sized) per day (Weir et al., 2012) and that the total surface area of the human small intestine is approximately 2×10^6^ cm^2^ (DeSesso and Jacobson, 2001), meaning that the amount of TiO_2_ NP ingested per cm^2^ of intestine is ∼10 ng cm^−2^. For *in vitro* studies, 25 µg cm^−2^ of TiO_2_ NP within a food matrix was used for cell exposures, which is 10^3^-10^4^ times more than the average adult intake and represents the worst-case scenario (Brun et al., 2014). The gut co-culture model was exposed to 25 µg of TiO_2_ NP cm^−2^ of food digest, or 25 µg cm^−2^ of TiO_2_-free food digests, serving as control, for 4 h (Brun et al., 2014; Weir et al., 2012). The cells exposed to TiO_2_ food digests showed no statistically significant change (*P*=0.45) in permeability across the monolayer, compared to the TiO_2_-free digests (Fig. 2A).

The *in vitro* experiments for exposure to HS were performed using a co-culture model of Caco-2 and HT29-MTX cells adapted to low glucose medium (5 mM). This concentration was used because a non-diabetic subject has a blood glucose concentration in the range of 4-8 mM (Wray, 2011). The high glucose concentration used *in vitro*, 25 mM glucose, is used because diabetic patients exhibit hyperglycemia, which is blood sugar above 10 mM (Wray, 2011). The control sample also included mannitol, which is structurally similar to glucose, but is unlikely to affect metabolism or permeability (Wong et al., 2009). The use of mannitol allows us to exclude potential hyperosmotic effects that may arise due to high glucose. A significant increase in gut permeability was observed in the samples exposed to HS (25 mM glucose), compared to the control cells (exposed to 5 mM glucose and 20 mM mannitol) (D'Souza et al., 2003), at both 2 h and 24 h following HS exposure (Fig. 2B,C).

### TWEEN 20 and salt diets significantly reduce lifespan

**Fig. 3.**
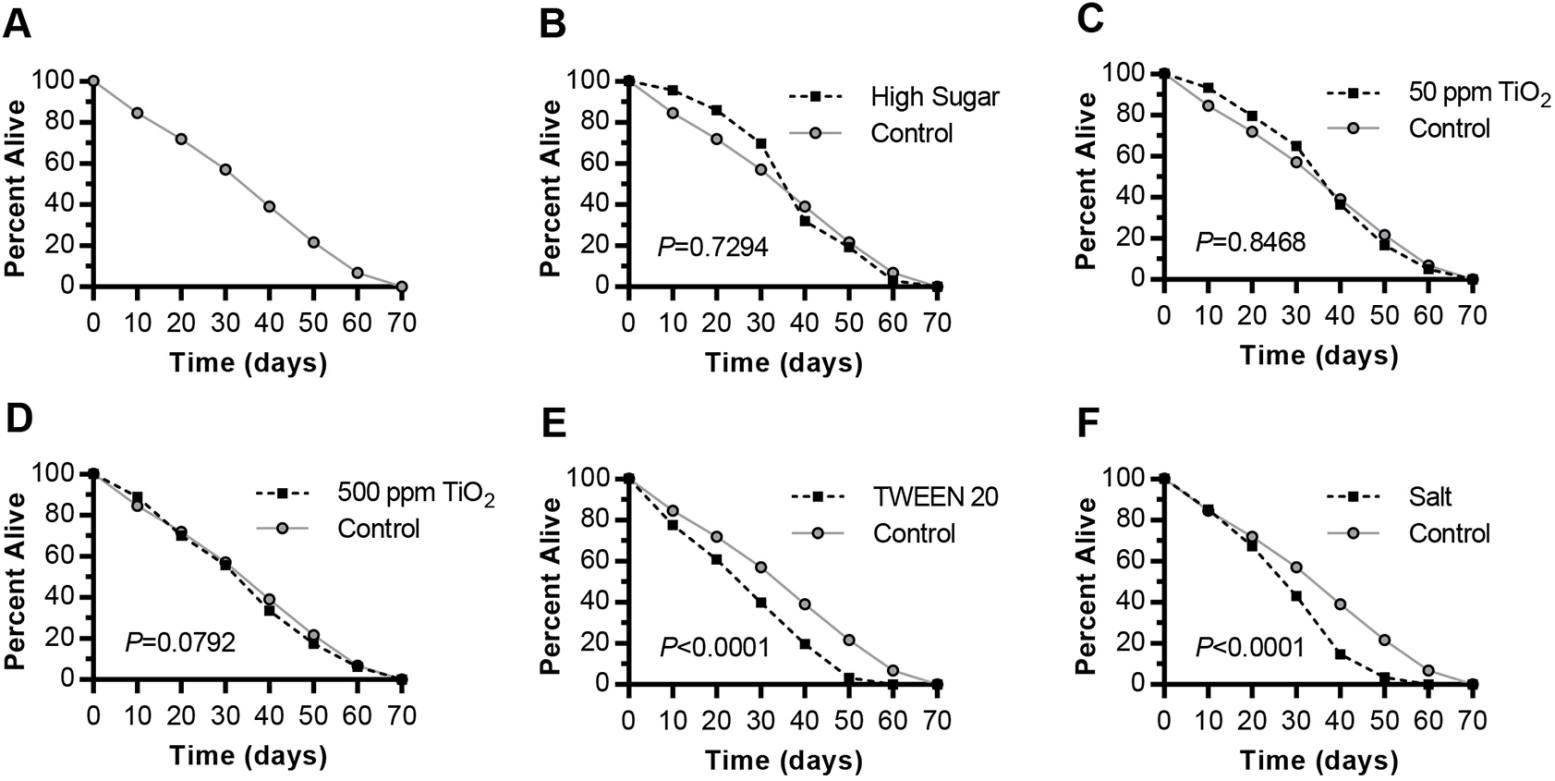
**Food-additive exposure reduces adult fly survival under some feeding conditions.** Each experimental diet was compared with the control diet. (A) Control diet (0.15 M sucrose; *n*=945). (B) High-sugar (HS) diet (1.0 M sucrose; *P*=0.7294, χ^2^ 0.1197, d.f.=1; *n*=797). (C) 50 ppm TiO_2_ diet (*P*=0.8468, χ^2^ 0.0373, d.f.=1; *n*=999). (D) 500 ppm TiO_2_ diet (*P*=0.0792, χ^2^ 3.082, d.f.=1; *n*=1189). (E) 1% TWEEN 20 diet (*P*<0.0001, χ^2^ 142.1, d.f.=1; *n*=937). (F) 1% salt diet (*P*<0.0001, χ^2^ 135.8, d.f.=1; *n*=949). *P*-values were calculated using the Mantel–Cox log-rank test.

Because previous studies showed that Smurfing is highly correlated with death, we analyzed fly survival on each diet. Although HS feeding had the greatest cumulative increase in Smurfing, it did not significantly reduce lifespan in this experiment (Fig. 3A,B). We note that the shape of our longevity curve is peculiar, with an unexpected degree of early lethality even on control diets (Fig. 3A), although we were careful to monitor food quality, to rear flies at low densities, to transfer them every 3-5 days and to remove from analysis any flies that appeared to have died due to sticky food conditions. Longevity curves are often more sigmoidal (Lushchak, 2014), although a more linear relationship has been seen in other studies (Moskalev et al., 2016). TiO_2_ NP did not have dramatic effects on either Smurfing or death (Figs 1C,D and 3C,D). Other supplemented diets showed a negative correlation between Smurfing and lifespan. TWEEN 20 and salt diets both led to significantly reduced longevity compared with control-fed flies (Fig. 3E,F), with survival curves showing earlier death in TWEEN-20-fed than salt-fed flies, which correlated with predominantly early (TWEEN 20)- versus late (salt)-age gut barrier dysfunction (Fig. 1K,L; Fig. S1C-K). These data are consistent with a previous study showing that increased Smurfing in flies reared on a higher-yeast diet correlated with a higher mortality rate, and suggest that the relationship between Smurfing and death depends upon the diet (Rera et al., 2012).

### Age and dietary sugar affect intestinal alkaline phosphatase activity

**Fig. 4.**
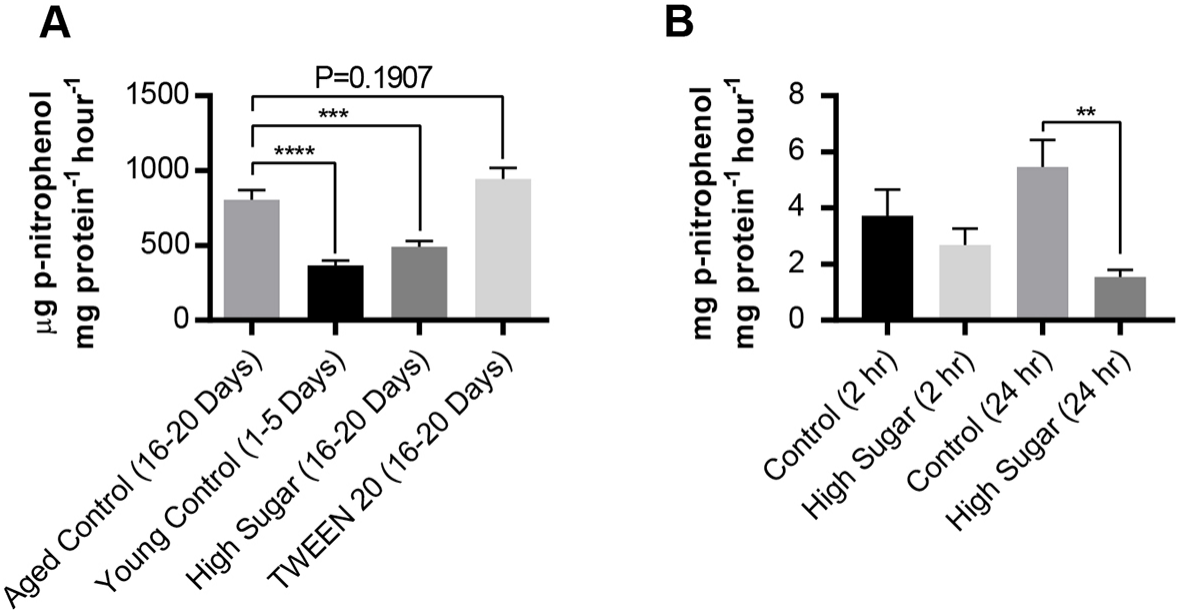
**Dietary supplementation affects intestinal alkaline phosphatase (IAP) activity in adult flies and intestinal cell co-cultures.** Alkaline phosphatase activity was tested using the substrate *p*-nitrophenol phosphate. (A) Gut lysates were tested after 3 weeks of feeding each additive (*n*=26 per diet). Error bars represent s.e.m. ***P<0.001; ****P<0.0001, compared to aged controls, using a one-way ANOVA. (B) Alkaline phosphatase assay for Caco-2/HT29-MTX cell monolayers exposed to control (5 mM glucose+20 mM mannitol) or high glucose (25 mM; HS) for 2 or 24 h (*n*=12). Error bars represent s.e.m. **P <0.01 using a two-tailed Mann–Whitney test.

Because IAP activity is associated with gut integrity in humans, we tested whether gut permeability was associated with IAP activity in *Drosophila*. For these studies, the HS and 1% TWEEN 20 diets were fed for 16-20 days because both led to a relatively high degree of Smurfing at this time point when compared with control diets (Fig. S1E). We also compared young and aged guts because Smurfing increases with age (Dambroise et al., 2016; Rera et al., 2012; Tricoire and Rera, 2015) and IAP activity varies (Huygelen et al., 2014; Jang et al., 2000; Welsh et al., 1985). Guts were isolated from adult females and assayed for IAP activity using an artificial substrate, *p*-nitrophenol phosphate (pNPP). Middle-aged (16-20 days old) flies exhibited higher IAP activity, compared with young (1-5 days old) flies (Fig. 4A), consistent with previous reports of dysbiosis upon aging (Huygelen et al., 2014; Jang et al., 2000; Welsh et al., 1985). Middle-aged flies reared on a HS diet exhibited significantly less IAP activity than middle-aged flies reared on control diets. Finally, 16- to 20-day-old flies reared on a 1% TWEEN 20 diet exhibited no significant difference in IAP activity compared to flies reared on a control diet (Fig. 4A), despite the significant effect of TWEEN 20 on Smurfing (Fig. 1E,K).

### Added sugar reduces phosphatase activity in human gut cell co-cultures

Like the *in vivo* model, the *in vitro* model was also assessed for IAP activity. The Caco-2/HT29-MTX cell monolayer was subjected to control (5 mM glucose+20 mM mannitol) or HS (25 mM glucose) conditions for 2 or 24 h (D'Souza et al., 2003). Similar to results *in vivo*, the average IAP activity for cells exposed to HS were decreased at both time points when compared to controls (Fig. 4B). However, statistical significance was only observed at the 24-h time point. These data suggest that there is a complex relationship between IAP and gut permeability in the *in vivo* and *in vitro* models.

### High sugar leads to numerous changes in gut structure and function

**Fig. 5.**
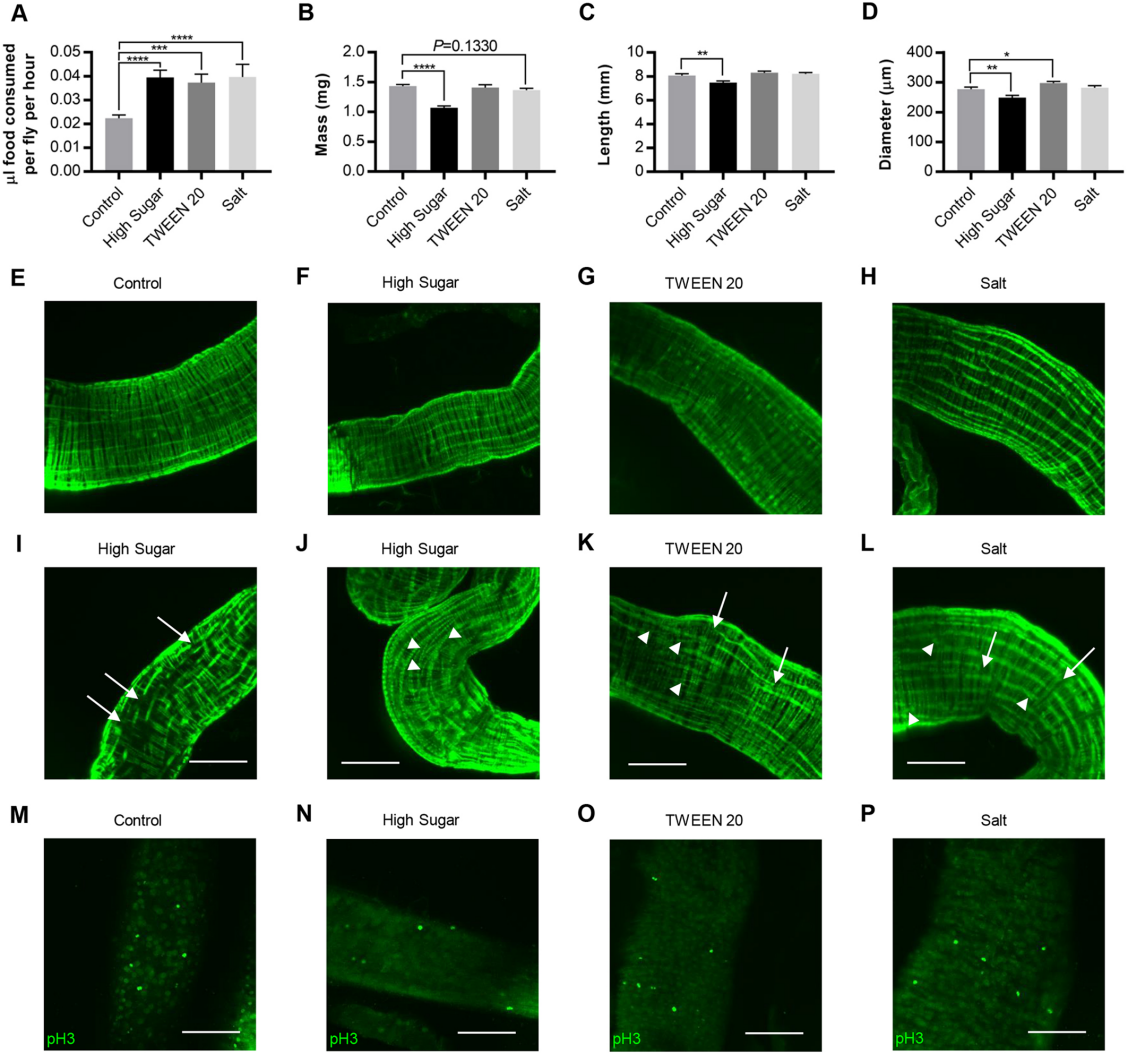
**The mechanisms by which HS and other additives affect gut function are likely to differ.** Females were aged 3 weeks and quantified for various phenotypes. (A) One-hour feeding volume. (B) Fly mass. (C) Midgut length. (D) Midgut diameter at its widest point, found in the posterior midgut. Each diet was compared with control using a Student's two-tailed *t*-test. Error bars represent s.e.m. *P<0.05; **P<0.01; ***P<0.001; ****P<0.0001. (E-H) Actin cytoskeleton of the posterior midgut, which is usually uniform and exhibits few breaks, dead ends or crossovers. (E) Control diet. (F) HS diet. (G) TWEEN 20 diet. (H) Salt diet. (I-L) Aberrant actin cytoskeletons found in additive guts. Arrows denote broken filaments and arrowheads denote filaments that differ from the typical parallel filament structure. (I) HS, filament breaks. (J) HS, filament disorganization and crossing over. (K) TWEEN 20. (L) Salt. (M-P) Anti-phospho-histone H3 immunoreactivity in posterior midguts. This region typically had 0 or 1 dividing cell per field, although some fields had five or more. (M) Control-fed posterior gut with numerous dividing cells. (N) A HS-fed gut. (O) Dividing cells in a TWEEN-20-fed gut. (P) Dividing cells in a salt-fed gut. Scale bars: 100 µm.

To better understand the mechanisms by which the diets affected gut function, we examined the feeding behavior and gut morphology of flies that consumed different diets. Three weeks of HS, TWEEN 20 or salt feeding significantly increased food intake to a similar degree (Fig. 5A). However, HS reduced overall adult weight (Fig. 5B), gut length (Fig. 5C) and gut diameter (Fig. 5D), whereas TWEEN 20 and salt did not. To further explore the mechanisms underlying increased gut permeability in HS and other permeability-increasing diets, we examined gut structure and cell proliferation at the cellular level. Gut structure seemed to be most strongly affected in the posterior (R4) midgut region, which plays a role in nutrient absorption, expressing high levels of lipid metabolic enzymes and transporters (Buchon and Osman, 2015) and undergoing significant cell division (Strand and Micchelli, 2013). A fluorescent phalloidin was used to visualize the actin cytoskeleton using confocal microscopy. Most guts had organized, continuous filaments running along the length of the midgut regardless of diet (Fig. 5E-H). Three weeks of HS, TWEEN 20 or salt feeding led to broken and disorganized actin filaments in a small number of posterior midguts (Fig. 5I-L). These effects were qualitatively striking compared with control feeding and compared with the anterior gut, which did not differ in actin among diets. Phospho-histone H3 immunoreactivity was used to detect cell division, which was more frequently observed in the posterior midgut compared with the anterior midgut, although the number of dividing cells per field varied widely and did not seem to differ significantly among diets (Fig. 5M-P).

### Bacterial exposure affects intestinal permeability *in vivo* and *in vitro*

**Fig. 6.**
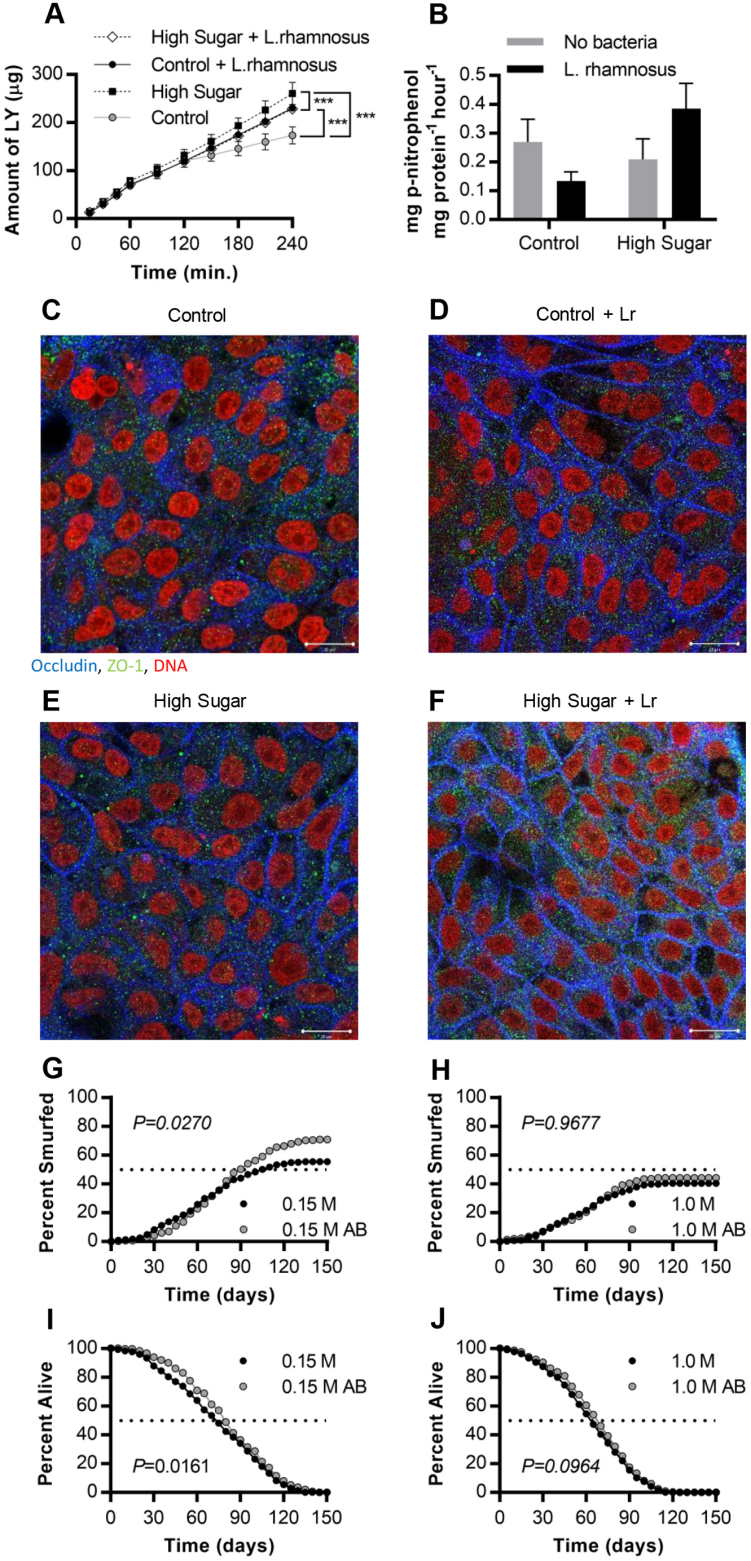
**The microbiome interacts with sugar to control gut function.** (A) Amount of Lucifer Yellow (LY) transferred across the Caco-2/HT29-MTX cell monolayer following exposure to control (5 mM glucose+20 mM mannitol) or high glucose (25 mM glucose; HS) with or without 10^3^ CFU ml^−1^ *L. rhamnosus* for 4 h (*n*=6). ***P<0.0001 using a non-linear fit for a linear model of each sample set. (B) Alkaline phosphatase activity for co-cultures exposed to control or HS and 10^3^ CFU ml^−1^ *L. rhamnosus* for 4 h (*n*=10). (C-F) Confocal images of the *in vitro* epithelium stained with immunofluorescence for occludin (blue), ZO-1 (green), integral plasma membrane proteins located at the tight junctions, and DNA (red) after 4 h exposure to (C) control (5 mM glucose+20 mM mannitol), (D) control (5 mM glucose+20 mM mannitol) and 10^3^ CFU ml^−1^ *L. rhamnosus* (Lr), (E) HS (25 mM glucose), (F) HS (25 mM glucose) and 10^3^ CFU ml^−1^ *L. rhamnosus*. Scale bars: 20 µm. (G-J) Adult *Drosophila* were fed a control or HS diet with or without antibiotics. (G) Cumulative Smurfing in flies fed the control lab diet +/− an antibiotic cocktail for the entire lifespan (*P*=0.0270, χ^2^ 4.891, d.f.=1; *n*=314 for control; *n*=302 for control+antibiotics). (H) Smurfing in flies reared on HS +/− antibiotics (*P*=0.9677, χ^2^ 0.001644, d.f.=1; *n*=304 for HS; *n*=314 for HS+antibiotics). (I) Lifespan after control rearing +/− antibiotics (*P*=0.0161, χ^2^ 5.792, d.f.=1; *n*=314 for control; *n*=302 for control+antibiotics). (J) Lifespan after HS +/− antibiotics (*P*=0.0964, χ^2^ 2.764, d.f.=1; *n*=304 for HS; *n*=314 for HS+antibiotics). Error bars represent s.e.m. *P*-values were calculated using the Mantel–Cox log-rank test.

To determine whether commensal bacteria play a role in sugar-dependent gut barrier dysfunction, we modulated the microbiome in the *in vitro* and *in vivo* models. We tested the role of *L.* *rhamnosus* GG (Gorbach–Goldin) in human gut epithelial co-cultures. The Caco-2 and HT29-MTX co-culture was exposed to control or HS conditions, with or without 10^3^ colony forming units (CFU) ml^−1^ *L. rhamnosus* for 4 h. This concentration was used because the epithelial cell viability decreases at bacterial concentrations higher than 10^3^ CFU ml^−1^ (Richter et al., 2018). Under control culture conditions, *L. rhamnosus* increased the permeability of the model; however, in combination with HS medium, *L. rhamnosus* caused a decrease in LY permeability when compared to epithelial co-cultures without bacteria (Fig. 6A). IAP activity of the co-cultures also seemed to depend upon *L. rhamnosus* in a glucose-dependent manner, although the effect was not statistically significant (Fig. 6B). Bacterial viability data showed an equal increase in growth of *L. rhamnosus* in low- and high-sugar conditions so that bacterial number did not differ (Fig. S2), although bacterial metabolism might have. To better understand the cause of change in intestinal permeability at the cellular level, the tight-junction proteins (occludin and ZO-1) were visualized using confocal microscopy. While the HS medium alone did not induce changes in occludin or ZO-1 expression (Fig. 6C,E), bacterial exposure resulted in an increase in ZO-1 in HS conditions (Fig. 6E,F). *Lactobacillus rhamnosus* also induced an increase in occludin expression (Fig. 6C,D).

In parallel, we took an antibiotic feeding approach to disrupt the gut microbiome *in vivo*. Smurfing for this experiment was recorded by dividing the total number of Smurfs (dead or alive) by the number of flies within the population, with each fly counted only once as in previous Smurf studies (Rera et al., 2012). For these studies, we used a conventional cornmeal-yeast laboratory diet, which is lower in protein and overall calories than the richer semi-defined food used earlier in this study and in our previous studies. This conventional medium increases lifespan and overall percentage of Smurfing compared with the rich medium and therefore has the potential to improve the Smurf assay's sensitivity (maximum lifespan of 130 days versus 70 days and cumulative Smurfing around 55% on conventional versus 14% on the rich diet). Antibiotic feeding led to increased gut permeability during control sugar feeding conditions (Fig. 6G) but not during HS feeding (Fig. 6H) on this conventional diet. Antibiotic feeding produced a minor but statistically significant increase in the lifespan of control-fed flies (*P*<0.05, Fig. 6I) and antibiotics produced a trend toward increased lifespan in HS-fed flies (*P*<0.1, Fig. 6J), although the magnitude of increase in lifespan was very small compared with the differences observed based on diet. Taken together, our studies are consistent with a model where the diet and microbiome interact to control gut function and overall animal health.

## DISCUSSION

In this work, we tested the effects of food additives on intestinal integrity using both *in vivo* and *in vitro* models. Dietary additives used at subtoxic and physiologically relevant levels increased permeability in both human and *Drosophila* multicomplex model systems. Our work suggests that the increasing concentrations of additives present in our food may be directly influencing the increasing prevalence of diseases associated with abnormal intestinal permeability. Of the diets we tested, the HS diet elicited the highest cumulative proportion of Smurfs on our rich medium. The HS exposure in the *in vitro* model also showed a significant increase in intestinal permeability (Fig. 2B,C). The *in vitro* increase may seem subtle until one considers the chronic exposure and size of the exposed small intestine (2×10^6^ cm^2^ compared with the 0.33 cm^2^ co-culture), making it likely that small differences would be magnified (Brun et al., 2014). High amounts of added sugar are being increasingly used in foods for their sweetness, texture and natural preservative properties (Manzel et al., 2014). Sugar-sweetened beverage consumption is associated with an increase in obesity, hypertension and other cardiometabolic diseases worldwide (Deshpande et al., 2017; Singh et al., 2015). Therefore, it is of interest to better understand the complex effects of dietary sugar on gut function.

Glucose and fructose primarily cross the gut epithelium by facilitated diffusion. Glucose is typically transported through sodium glucose cotransporter 1 (SGLT1) (Hopfer et al., 1973; Röder et al., 2014; Uhing and Kimura, 1995), and fructose through glucose transporter 5 (GLUT5) (Barone et al., 2009; Kayanos et al., 1990). The transport of these sugars has been shown to recruit additional SGLT1 and GLUT5 proteins to the plasma membrane as well as trigger conformational changes in tight junctions in order to enhance transport (Madara and Pappenheimer, 1987; Madara et al., 1987, 1993; Pappenheimer, 1987; Pappenheimer and Reiss, 1987; Sadowski and Meddings, 1993). Another study proposed that glucose transport triggers the recruitment of GLUT2 to the apical surface to enhance transport even further (Gouyon et al., 2003). The increasing number of transporters recruited as absorption occurs supports another finding that over 90% of glucose transport occurs transcellularly (Uhing and Kimura, 1995). Hyperglycemic, insulin-resistant mice exhibit increased intestinal permeability and infection susceptibility, both of which are GLUT2 dependent (Thaiss et al., 2018). In addition to potential direct effects of sugar on transport, HS diets have been shown to generate intestinal stress, decrease gut size, increase reactive oxygen species (ROS) and cell death, and disrupt cell differentiation and morphology in flies (Zhang et al., 2017). Interestingly, hyperglycemia was also associated with Smurfing in flies that experienced traumatic brain injury (Katzenberger et al., 2015). We propose that the HS diet shifts gut metabolic homeostasis in a manner that compromises epithelial integrity. Feeding increased with all additive diets, suggesting that nutrient availability is high, although nutrient absorption may be diminished, especially on the HS diet, which reduced both gut and animal size. Consistent with this, we observed some structural changes upon sugar overload in both the fly gut and the human co-culture model. In whole-mounted fly guts, actin filaments seemed to be disrupted. *In vitro*, high glucose has been shown to decrease transepithelial resistance, diffuse the distribution of actin filaments and decrease the transepithelial transport of solutes (D'Souza et al., 2003). Paracellular transport occurs in adult flies upon cold stress (MacMillan et al., 2017). At the cellular level, excess sugar may overcome the threshold of transporters at the apical surface, leading to an excess of sugars to the crypts at the roots of the villi where tight junctions reside, pushing the remaining sugars to be absorbed paracellularly. Because tight junctions do not normally withstand this magnitude of traffic, it is possible that increased permeability causes wear and tear on the junction, eventually leading to loss of integrity and failure, or even cell death.

Contrary to a previous report (Rera et al., 2012), we did not find that all flies Smurfed before death. One possible reason is that, due to the richness of our experimental diets, flies may not have ingested enough blue dye to enable detection of Smurfing in all flies. In addition, older flies eat less than younger flies (Wong et al., 2009), which may have reduced sensitivity and allowed us to detect only the most severe gut barrier dysfunction in older flies. It is also possible that some experimental diets lead to death in a manner that is gut independent. Nonetheless, Smurfing and death were positively correlated in five of the six diets, with the exception of HS diets, which increased Smurfing without significantly affecting lifespan. TWEEN 20 and salt feeding led to increased Smurfing and reduced longevity, whereas control and TiO_2_ diets produced the least amount of Smurfing and had the highest overall longevity of the diets tested (Figs 1A,C-F and 3A,C-F). The different diets seemed to correspond with different rates of Smurfing over time, suggesting that there may be different mechanisms by which each diet affects the gut. Smurfing occurred earlier on HS and TWEEN 20 diets, in contrast to what tended toward greater significance for later time points in salt-fed Smurfs (Fig. 1H,K,L; Fig. S1C-K). In general, the percentage of dead flies who were Smurfs declined as the population aged. Of note, HS feeding reduced longevity without increasing gut permeability compared with control feeding in our low-protein, reduced-calorie fly dietary medium (Fig. 6G-J). These data suggest that early death may be more likely to be associated with increased gut barrier dysfunction on certain stressful diets.

HS feeding resulted in reduced IAP activity compared with controls. IAP is a gut mucosal defense factor that detoxifies bacterial LPS by dephosphorylation of the lipid A moiety (Beumer et al., 2003; Poelstra et al., 1997), thereby protecting against LPS-induced inflammation (Goldberg et al., 2008). IAP also regulates bicarbonate secretion and duodenal surface pH (Akiba et al., 2007; Mizumori et al., 2009), and both regulates and is regulated by intestinal lipid uptake across the enterocyte membrane (Guthmann et al., 2002; Hansen et al., 2007; Narisawa et al., 2003). We did not observe a strict correlation between IAP activity and Smurfing (Figs 1A,B,E,G,H,K and 4A). However, aging and diet do both affect gut barrier function and IAP activity, suggesting that a complex relationship may exist between them. IAP sits at a nexus of biochemical and inflammatory pathways in the intestinal lumen that are known to vary with age (Huygelen et al., 2014; Jang et al., 2000; Welsh et al., 1985) and luminal contents (Bamba et al., 1990; Goldberg et al., 2008; Montoya et al., 2006; Sogabe et al., 2004, 2007). Age increased IAP function in adult flies (Fig. 4A), which could relate to the increased dysbiosis (Huygelen et al., 2014; Jang et al., 2000; Welsh et al., 1985) or to the reduced nutrient intake (Morley, 2001) that have been shown to occur with aging. IAP can also promote fatty acid uptake (Guthmann et al., 2002; Mizumori et al., 2009), which the gut might decrease when carbohydrates are plentiful in HS conditions. Sugars are a more flexible energy source than fatty acids, enabling the biosynthesis of amino acids and nucleotides in addition to fat, and might therefore be preferred. Interestingly, HS and TWEEN 20 diets had different effects on IAP, and therefore may affect gut permeability via different mechanisms.

Our results with the surfactant TWEEN 20 also coincide with what is known about the effects of these surface-tension-lowering compounds on gut permeability. TWEEN 20 has been shown to increase paracellular permeability both *in vivo* and *in vitro* (Al-Saraf et al., 2016; Kang et al., 2012; Lo, 2003). Surfactants are often used in oral drug delivery as they can remain stable long enough for the drug to reach the intestine, where the surfactant can act on the epithelium, increasing permeability (Rong Liu, 2008). Many of the surfactants used in drugs to increase absorption are also being used in foods as mixing agents or emulsifiers (Rong Liu, 2008). The ability of TWEEN 20 to disrupt protein-lipid interactions is likely to be the cause for its acceleration of intestinal barrier dysfunction as well as mortality (Figs 1E and 3E). The increased mortality and Smurfing of flies chronically fed HS and TWEEN 20 diets is consistent with a published report that the loss of intestinal barrier function leads to death (Rera et al., 2012). Unlike HS feeding, we did not observe biochemical effects or reduced size in flies fed TWEEN 20 diets, compared with control-fed flies (Figs 4A and 5B). The mechanisms underlying increased gut permeability are therefore likely to differ between sugar- and emulsifier-induced barrier dysfunction.

Commonly used as a white dye and a texture-modifying additive, TiO_2_ NP can be found in foods, adhesives, paints and toothpaste. Several studies have been conducted to assess the role of TiO_2_ NP on the intestinal membrane; however, brush-border disruption due to TiO_2_ from human food products is mostly an unexplored field (Bettini et al., 2017; Faust et al., 2014). Using food-grade TiO_2_, and subjecting it to *in vitro* digestion, aids in observing the effects of ingested TiO_2_. TiO_2_ NP had minor and variable effects on intestinal permeability and survival when tested using our *in vivo Drosophila* feeding conditions (Figs 1C,D and 3C,D). This is consistent with previous work performed by our research group where TiO_2_ affected gut function primarily through microvilli remodeling (Guo et al., 2017; Richter et al., 2018). Similar results were seen in the *in vitro* model, with no change in permeability upon exposure to TiO_2_ (Fig. 2A). Still, TiO_2_ NP do affect gut morphology and physiology in rats, altering immune cell populations, cytokine levels and crypt morphology after chronic exposure (Bettini et al., 2017). Future studies may explore the role of TiO_2_ in infection and disease models that are likely to be more sensitive to NP.

Sodium is typically absorbed across the gut epithelium down its concentration gradient via cotransporters, including SGLTs (Röder et al., 2014). SGLTs aid in glucose transport by recruiting canonical glucose transporters (Kellett and Brot-Laroche, 2005), triggering contraction of perijunctional actomyosin (Madara and Pappenheimer, 1987) and by creating an osmotic driving force for paracellular glucose flow through tight junctions (Madara and Pappenheimer, 1987; Pappenheimer and Reiss, 1987). For these reasons, sodium ions can increase the rate of glucose uptake (Hopfer et al., 1973). *Drosophila* have conserved NaCl-sensing sodium/solute cotransporters in what is known as the SLC5 protein family, which control Malpighian (renal) tubule physiology, although no role has yet been described for these proteins in intestinal glucose transport (Dus et al., 2013; Stergiopoulos et al., 2009). Salt guts exhibited aberrant actin filament structure similar to the HS diet in a fraction of the posterior midguts analyzed. Although sodium chloride and TWEEN 20 led to similar levels of overall Smurfing (16.5% and 22.8%, respectively; Fig. 1E,F), the ages at which gut barrier function seemed to be strongly affected varied (Fig. 1K,L; Fig. S1C-K). This suggests that there may be different mechanisms underlying the increases in permeability. Salt-fed flies exhibited higher lethality than control-fed flies and died before reaching the age of 60 days (Fig. 3F). We may see increased death with salt due to a disruption in protein-protein interactions, which could enable increased paracellular leakiness and transmigration of detrimental bacteria across the epithelium. Together, these studies demonstrated that feeding salt and other biologically active ingredients compromise gut barrier function in a common laboratory wild-type *Drosophila* genotype.

Diet has been shown to affect the composition of the human and mouse gut microbiota, which in turn can affect susceptibility to infection (Guan et al., 2016; Zackular et al., 2016), as well as other aspects of health (Kabeerdoss et al., 2012; Matijašić et al., 2014). Studies have shown that increased bacterial load is closely associated with intestinal barrier dysfunction (Cheng et al., 2013; Clark et al., 2015; Rera et al., 2012). One study showed that clearing the microbiota from *w^1118^* flies who experience barrier dysfunction significantly increased lifespan (Brummel et al., 2004), although another study showed no effect of microbiota clearance on lifespan in another genotype, *Oregon-R* (Ren et al., 2007). We found that the gut microbiome's effect depended upon diet. Under HS conditions, antibiotics had little effect on the measured traits, whereas, under control conditions, the microbiome seemed to play a role in both gut function and survival. Interestingly, antibiotic feeding increased Smurfing but also increased survival, indicating that the endogenous laboratory microbiome plays both beneficial and deleterious roles under the conditions tested. Therefore, barrier dysfunction alone may not lead directly to death, but instead gives the microbiota the opportunity to infect the host by crossing the epithelium, which in turn results in death (Clark et al., 2015).

Under normal conditions, occludin and ZO-1 play regulatory and structural roles in the small intestinal tight junctions. *Lactobacillus rhamnosus*, due to its probiotic properties, restores occludin in a compromised gut (Zhao et al., 2015). Increased occludin expression in the presence of *L. rhamnosus* is recreated in the *in vitro* model (Fig. 6D). ZO-1 is a part of the innate immunity of the gut, and a previous study with mice showed that inappropriate upregulation of ZO-1 correlates with increased intestinal permeability and progression towards type 1 diabetes (Watts et al., 2005). Similarly, *in vitro* results with HS and *L. rhamnosus* show an increase in permeability (Fig. 6A) and an upregulation of ZO-1 (Fig. 6F), further suggesting the involvement of commensal bacteria in the regulation of the intestinal barrier. Taken together, our studies suggest that flies and humans have remarkably similar gut physiology, and highlight the complex relationship between diet and gut function.

## MATERIALS AND METHODS

### Fly stocks

The standard laboratory strain *w^1118^* was purchased from the Vienna Drosophila Resource Center and was used for all *Drosophila* experiments. Newly eclosed females were maintained with males for 3 days before segregation into female-only vials for aging.

### Fly diet preparation

Fly stocks were maintained on standard lab food containing yeast, 5% dextrose, cornmeal and agar. For all experiments except the antibiotic feeding experiments, the control diet was a modified Bloomington semi-defined medium containing 5% sucrose, 8% yeast, 2% yeast extract, 2% peptone and 1% agar (Musselman et al., 2011). FD&C blue dye #1 was added at a concentration of 2.5% (wt vol^−1^) to allow detection of the Smurf phenotype (Rera et al., 2012). Experimental diets were prepared by either adding 1% NaCl (w vol^−1^), 1% TWEEN 20 (vol vol^−1^), 50 ppm or 500 ppm TiO_2_ NP (30 nm anatase, US Research Nanomaterials, Inc.), or increasing the sucrose concentration from 5% to 34% (wt vol^−1^) to produce a 1 M high-sugar (HS) diet. For antibiotic feeding experiments, the control diet was the standard lab cornmeal-agar medium using 5% or 34% dextrose, 0.9% agar, 3.5% yeast and 8% cornmeal, and 2.0% blue dye was used instead of 2.5%. Tetracycline and ampicillin were mixed into the food after cooling at final concentrations of 50 µg ml^−1^ and 500 µg ml^−1^, respectively (Brummel et al., 2004). After the food had solidified, 100 µl of 10,000 units ml^−1^ penicillin and 10 mg ml^−1^ streptomycin was added to the top of each antibiotic food vial (Pueyo et al., 2016).

### Smurf and survival assays

Adult female flies were reared on either control blue food or one of the experimental additive blue diets. Live flies were anesthetized with CO_2_ and visually scored for the Smurf phenotype every 5-7 days until all flies died, providing the percent of flies Smurfed by each diet during each time interval. Alongside visual scoring, vials were checked daily for dead flies, which were removed, washed and analyzed for the Smurf phenotype in order to record the cumulative percentage of flies Smurfed before death, as well as how long each fly survived on each food type. Each data point represented one fly. A fly was considered a Smurf when blue dye was found outside of the gut throughout the head, thorax, abdomen and legs (Fig. S1B and Rera et al., 2012). Male *Drosophila* exhibit reduced feeding and are difficult to score for Smurfing (Regan et al., 2016), and were therefore not used.

### Alkaline phosphatase assay in isolated adult *Drosophila* guts

Female adult flies were anesthetized using CO_2_ in groups of 5 for each biological replicate. The fly was placed onto a glass slide and submerged in phosphate-buffered saline (PBS) during dissection. Using forceps, the entire GI tract was extracted from the body by gently grasping the thorax, while pulling at the bottom portion of the abdomen. The foregut, hindgut, and Malpighian tubes were removed. The midgut was immediately transferred into a tube containing 100 µl PBS at −20°C prior to being stored at −80°C. For assay, midguts were homogenized using a motorized pestle. Twenty-five microliters of homogenate were added to 175 μl alkaline phosphatase assay reagent (Sigma N1891) in a 96-well plate and immediately read using an optical density (OD) wavelength of 405 nm. After a 1-h incubation at room temperature, the plate is read again. The OD at time zero is subtracted from the OD at 1 h in order to determine the rate of catalysis. Enzyme activity was normalized to total protein using Bradford's reagent (Amresco M172).

### Fly weights

Flies were raised on control food or one of the experimental foods for 3 weeks before being anesthetized and weighed in groups of 5 flies.

### Fly feeding experiments

Flies were raised on control food or one of the experimental foods for 3 weeks without FD&C blue dye #1 before being transferred to the same diet with blue dye from the hours of 4:00 PM-6:00 PM, which was the 2-h period at which the most food was consumed. After 2 h, flies were anesthetized and collected in groups of 4 to measure the amount of blue dye consumed. Flies with blue on the outside of the body were not used. Flies were homogenized in 200 μl of PBS buffer and centrifuged. The absorbance of the supernatant was measured at 630 nm using a microplate spectrophotometer (Molecular Devices) similar to Wong et al. (2009). The amount of blue dye in the samples was calculated from a standard curve made by serial dilution of blue dye in PBS buffer along with homogenate from age-matched flies exposed to non-dyed food in order to correct for absorbance of homogenate alone.

### Fly midgut measurements

Guts were collected from flies fed each diet for 3 weeks, placed on slides in groups of 3 and imaged immediately. Guts were imaged using a Jenoptik camera mounted on an Olympus SXZ10 stereomicroscope and then measured from the anterior tip of the proventriculus to the end of the midgut at the midgut-hindgut junction where the Malpighian tubules protruded. Measurements were taken using Progres Gryphax microscopy software calibrated on a hemocytometer.

### Fly immunohistochemistry

Fly guts were collected as described for gut measurements. Guts were fixed in 4% paraformaldehyde in PBS and Alexa-Fluor-488–phalloidin (Life Technologies A12379; 1:500) was used to visualize actin filaments. For phospho-histone H3, guts were fixed and stained as described previously (Micchelli and Perrimon, 2006) using an antibody that recognizes phospho-serine 10 (Sigma #H0412; 1:200). Secondary antibodies used were Alexa-Fluor-488 anti-rabbit IgG (Jackson ImmunoResearch 111-545-144; 1:250) and guts were whole-mounted using Vectashield (Vector Labs).

### Intestinal cell co-cultures

The human colon carcinoma Caco-2 cell line was purchased from American Type Culture Collection at passage 17 and used for experimentation at passage 45-55. The HT29-MTX intestinal epithelial cell line was kindly provided by Dr Thécla Lesuffleur of INSERM U560 in Lille, France, at passage 11 and used in experiments at passage 40-50 (Lesuffleur et al., 1990). Both cell types were grown in Dulbecco's modified Eagle medium (DMEM; Thermo Fisher Scientific) containing 4.5 g l^−1^ glucose and 10% (v v^−1^) heat-inactivated fetal bovine serum (HI-FBS; Thermo Fisher Scientific). The cells were maintained in a 5% CO_2_ incubator at 37°C and medium was changed every alternate day. Once the cells reached 80% confluency, they were passaged and seeded onto polycarbonate, 0.4 µm pore size, 0.33 cm^2^ membrane, 24-well Transwell inserts for permeability studies, or onto 24-well culture plates for IAP activity assays. Membranes or wells were coated with rat-tail type I collagen (BD Biosciences) at a concentration of 8 μg cm^−2^ for 1 h at room temperature. Cells were stained with Trypan Blue, counted, and seeded at a density of 100,000 cells cm^−2^ and a ratio of 75:25 (Caco-2:HT29) to allow for the formation of a mucus layer (Mahler et al., 2009). The cells were grown for 14 days before experiments were conducted. For the HS study, both cell lines were adapted to low-glucose medium by culturing for 5 passages in no-glucose DMEM (Thermo Fisher Scientific) supplemented with 5 mM sterilized glucose and 10% (v v^−1^) HI-FBS. Following adaptation to low-glucose conditions, cells were seeded as described above into Transwells or culture plates. Transepithelial resistance (TER) was measured prior to all permeability assays using an EVOM2 with the Endohm-6 chamber from World Precision Instruments. The Endohm chamber was soaked in 70% ethanol for 15 min, then 2 ml of sterile 100 mM KCl solution was added to the chamber, and it was connected to the EVOM2. A sterilized Calicell (WPI) with 200 µl KCl was then used to calibrate the chamber. Finally, the Endohm chamber was rinsed with sterile 18 MΩ water and equilibrated with 2 ml serum-free DMEM for 15 min before TER was measured for each Transwell membrane. Membranes with a TER of 250-300 Ω*cm^2^ were used for experiments.

### Bacterial cell culture

*Lactobacillus rhamnosus* and Caco-2/HT29-MTX tri-culture methods have previously been described (Richter et al., 2018). Briefly, *Lactobacillus rhamnosus GG* (*L. rhamnosus*) was purchased from American Type Culture Collection (ATCC 53103). *Lactobacillus rhamnosus* stock was serially diluted in 0.9% saline solution, plated on MRS agar (Difco^TM^) and allowed to grow in a 5% CO_2_ incubator at 37°C for 24 h. The OD of each dilution was quantified at a wavelength of 600 nm. To correlate absorbance with concentration (CFU ml^−1^), CFUs of *L. rhamnosus* at each dilution were counted and plotted to make a growth curve. Prior to each experiment, bacterial concentrations were determined using OD and added to the apical chamber of the Transwells in high- or low-glucose DMEM at a concentration of 10^3^ CFU ml^−1^.

### *In vitro* TiO_2_ nanoparticle food sample preparation

Cell cultures were exposed to TiO_2_ NP within a food matrix. M&M chocolate candies have been reported to be one of the top 10 products with the highest TiO_2_ content, and the hard outer shell serves as the source for TiO_2_ NP (Weir et al., 2012). M&M chocolate candies contain 1.25 µg of TiO_2_ mg^−1^ (Weir et al., 2012). Therefore, 6.6 mg M&Ms or 8.25 µg TiO_2_ in a 0.33 cm^2^ well was used to produce a dose of 25 µg cm^–2^ exposure in each Transwell. Controls were 6.6 mg well^−1^ of candy without the shell. Since the hard outer shells of M&Ms contain artificial colors as well, an equal number of M&Ms of each color were used to maintain consistency across digests.

The M&M samples were freeze-dried for 72 h, to remove all the water content from the food products, and then digested *in vitro* using the method first described by Glahn et al. (1998). The freeze-dried M&Ms were crushed with a mortar and pestle, and the weighed samples were subjected to ‘gastric’ digestion by adding 20 ml of 140 mM NaCl, 5 ml KCl, pH 2 solution to each sample and readjusting each sample to pH 2 with 1 M HCl. An aliquot of 1 ml porcine pepsin solution (25 mg ml^−1^, 800-2500 units mg protein^−1^, Sigma-Aldrich) in 0.1 M HCl was added to each sample, and the samples were rocked at 55 oscillations min^−1^ on a rocking platform for 1 h at 5% CO_2_ and 37°C. After the 1 h incubation, the pH of the samples was raised to 5.5-6.0 with 1 M NaHCO_3_, and 5 ml of porcine pancreatin and bile solution consisting of 2 mg ml^−1^ pancreatin (a mixture of trypsin, amylase, lipase, ribonuclease and protease activities; Sigma P3292) and 11 mg ml^−1^ bile extract (a mixture of glycine and taurine conjugates of hyodeoxycholic and other bile acids; Sigma B8631) was added. The pH was then adjusted to 7.0 with 1 M NaHCO_3_ and the volume of each tube was brought to 30 ml by weight with 140 mM NaCl, 5 mM KCl, pH 6.7. The samples were then referred to as digests and used for exposure studies.

### Food digest exposure

The Transwell plates were prepared for exposure by removing the medium from the wells and washing them with 1× PBS. A total of 200 μl of prepared TiO_2_ or TiO_2_-free digest was added to the apical chamber and 600 μl DMEM was added to the basolateral chamber. The cells were allowed to incubate with the digest for 4 h.

### High-glucose exposure

The Transwell plates seeded with low-glucose cells were first washed with 1× PBS. The sample wells were exposed to 200 μl of HS (25 mM glucose) solution in the apical chamber while the basolateral chamber was filled with 600 μl of 5 mM glucose DMEM. For the control wells, 200 μl of control solution (5 mM glucose+20 mM mannitol) was added to the apical chamber and 600 μl of 5 mM DMEM was added to the basolateral chamber. The short-term exposure was continued for 2 h, while the long-term exposure continued for 24 h. Post-exposure, 50 μM LY was added to the apical chamber of each of the wells and a permeability study was conducted as described below.

### Lucifer Yellow permeability study

A total of 100 μl of 50 μM LY was added to the apical chamber of the Transwell 1 h after addition of TiO_2_ or TiO_2_-free food digests. Samples (100 μl) were taken from the basolateral chamber of each well every 30 min for 4 h and replaced with 100 μl fresh culture medium. The samples were transferred to a 96-well opaque black-bottom plate. At the end of the exposure experiment, the 96-well plate was read using a Synergy 2 plate reader, controlled by Biotek's Gen5™ Reader Control and Data Analysis Software. The resulting fluorescence values were converted to amount of LY using a LY standard curve.

### Alkaline phosphatase activity for *in vitro* cell model

For IAP quantification, IAP and Bradford assays were performed on the cells grown in 24-well plates as previously described (Guo et al., 2017). The wells were washed with 1× PBS and 200 μl 18 megohm*cm deionized water was added to each well. The plate was sonicated at 4°C for 5 min before transferring the content of each well into different 0.5 ml microcentrifuge tubes. For IAP activity, 85 μl of pNPP solution was added to the required number of wells of a 96-well clear-bottom opaque side plate. To this, 25 μl of prepared samples and IAP standards (prepared using *p*-nitrophenol and pNPP) were added and allowed to incubate at 37°C for 1 h. For the Bradford assay, 250 μl of Bradford reagent was added to the required number of wells of a 96-well clear-bottom opaque side plate before transferring 5 μl of prepared sample or standards to those wells. The Bradford assay plate was incubated at room temperature for 30 min. The IAP and Bradford assay plates were read at 405 nm and 595 nm, respectively, using a Synergy 2 plate reader, controlled by Biotek's Gen5™ Reader Control and Data Analysis Software.

### Co-culture bacterial viability assay

A 100 µl sample of bacteria in culture medium or food digest was taken from the apical Transwell chamber post-permeability study. Bacteria were serially diluted in 0.9% sterile saline down to 10^−8^. A total of 10 µl of each dilution was plated in triplicate on MRS agar plates using a drop plate method. Plates were incubated at 37°C overnight and CFUs were counted.

### Co-culture immunohistochemistry

Caco-2 and HT29-MTX cells were grown on sterile coverslips in 6-well plates. After 14 days, the cell monolayers were exposed to control (5 mM glucose+20 mM mannitol) or HS (25 mM glucose) and 0 or 10^3^ CFU ml^−1^ *L. rhamnosus* for 4 h. Cells were fixed with 4% paraformaldehyde for 15 min, then incubated with 0.2% Triton X-100 in PBS for 10 min to permeabilize. Cells were blocked with 1% bovine serum albumin in PBS for 1 h at 4°C, washed, exposed to mouse anti-occludin (Invitrogen, # 33-1500, 1:100 in PBS), and rabbit anti-zonulin-1 primary antibodies (Abcam, ab59720, 1:100 in PBS) for 1 h at 4°C. The cells were then incubated in the dark for 2 h at room temperature with Alexa-Fluor-488 goat anti-mouse (Thermo Fisher Scientific # A32723, 1:100 in PBS) and Alexa-Fluor-568 goat anti-rabbit (Thermo Fisher Scientific # A11011, 1:100 in PBS) secondary antibodies. To stain DNA, DRAQ5 (Thermo Fisher Scientific) was used at 1:1000 in PBS. The cells were rinsed and the coverslips were mounted on glass slides with ProLong Gold mounting medium (Thermo Fisher Scientific) and cured overnight in the dark. Cells were imaged with a Zeiss LSM 880 NLO.

### Statistical analysis

Smurfing and survival comparisons of dead flies (Figs 1A-F, 3 and 6G-J) were performed using Kaplan–Meier estimation curves with the Mantel–Cox log-rank test. For the LY permeability study (Figs 2 and 6A), the curves were analyzed using a nonlinear fit test for a linear model. The variability in the data was removed using Box–Cox transformation and *P*-values were obtained by an *F*-test. The fly alkaline phosphatase assays were compared using a one-way ANOVA (Fig. 4A). The co-culture alkaline phosphatase assays and bacterial viability were analyzed using a two-tailed Mann–Whitney test (Figs 4B and 6B; Fig. S2). Fly weight and gut measurements, as well as Smurfing in live flies (Fig. 5A-D; Fig. S1C-K), were compared using a Student's two-tailed *t*-test. Statistical analysis was performed using GraphPad Prism 5 and differences were considered significant at *P*<0.05.

## Supplementary Material

Supplementary information
