# Supplementary material for: Effect of dietary additives on intestinal permeability in both Drosophila and a human cell co-culture
Source: Dis Model Mech. 2018 Nov 28;11(12):dmm034520. doi: 10.1242/dmm.034520 (PMC6307910; doi:10.1242/dmm.034520)
Supplement: Supplementary information [file dmm-11-034520-s1.pdf]

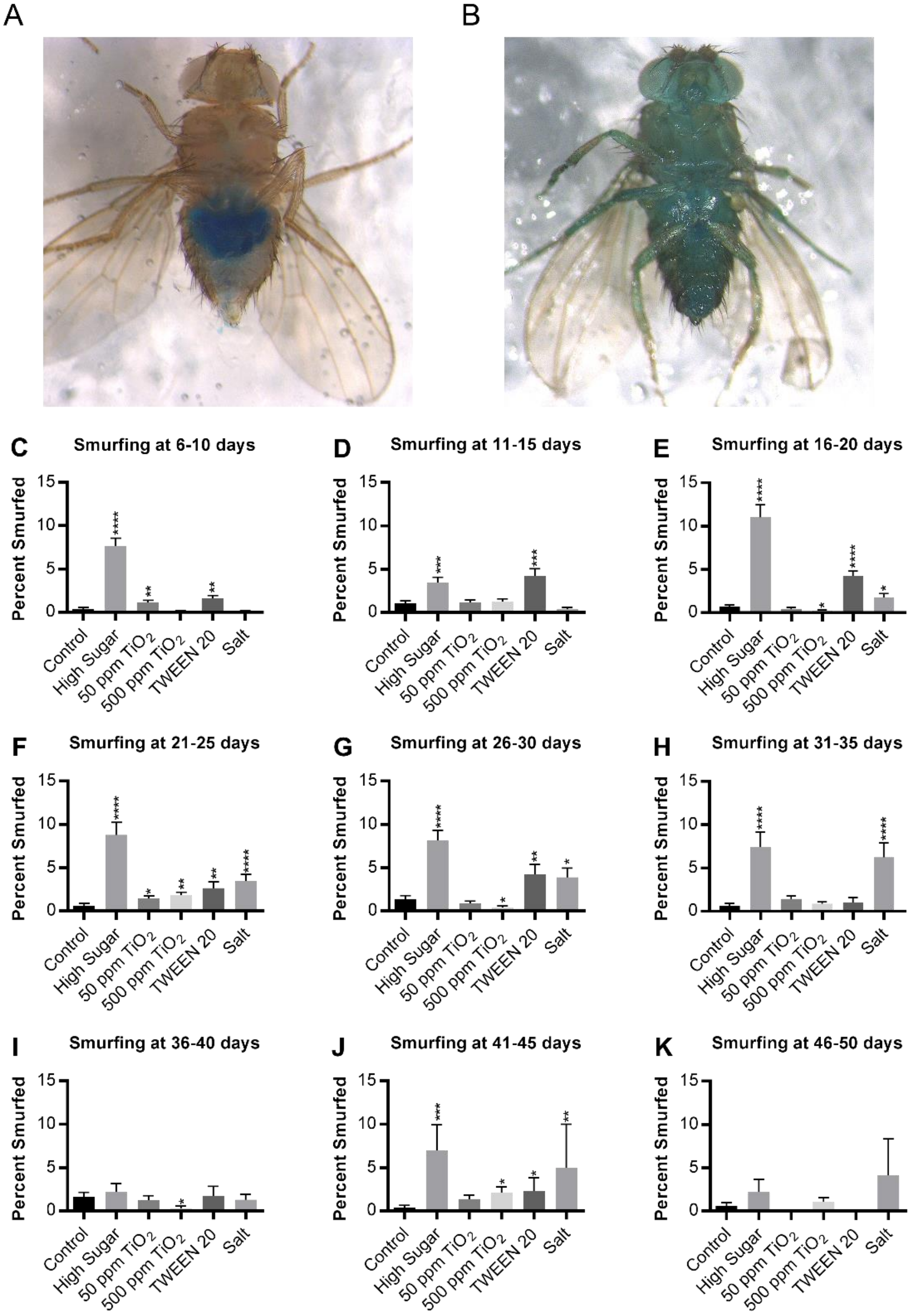

Figure S1. The Smurf phenotype's severity varies with age and diet in live flies. Photo of a non-Smurfed fly (A), where blue dye can be seen restricted to the gut in the anterior abdomen, and a Smurfed fly (B), where blue dye can be seen throughout the thorax, abdomen, legs, and in the head of the fly. (C-K) The percentage of live Smurfs observed throughout the lifespan on each diet. Each diet was compared with the control diet using a student's two-tailed *t* test. Error bars represent s. e. m. ***P* <0.01; ****P* <0.001; *****P* <0.0001.

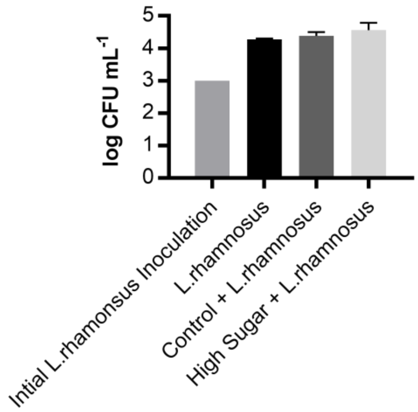

Figure S2. Bacterial viability post exposure to high glucose. Bacterial viability was assessed by drop plate method after 4 hour exposure of the in vitro intestinal model to control (5 mM glucose + 20 mM mannitol) or high sugar (25 mM glucose) and 10^3 CFU/mL *L. rhamnosus* (n=3). Error bars represent s. e. m., analyzed using a two-tailed Mann-Whitney test.

Disease Models & Mechanisms • Supplementary information
